# Efficacy of Selective PDE4D Negative Allosteric Modulators in the Object Retrieval Task in Female Cynomolgus Monkeys (*Macaca fascicularis*)

**DOI:** 10.1371/journal.pone.0102449

**Published:** 2014-07-22

**Authors:** Jane S. Sutcliffe, Vahri Beaumont, James M. Watson, Chang Sing Chew, Maria Beconi, Daniel M. Hutcheson, Celia Dominguez, Ignacio Munoz-Sanjuan

**Affiliations:** 1 Dept. of Neuroscience and CNS Safety Pharmacology, Maccine Pte Ltd, Singapore, Singapore; 2 CHDI Foundation/CHDI Management Inc., Los Angeles, California, United States of America; INSERM/CNRS, France

## Abstract

Cyclic adenosine monophosphate (cAMP) signalling plays an important role in synaptic plasticity and information processing in the hippocampal and basal ganglia systems. The augmentation of cAMP signalling through the selective inhibition of phosphodiesterases represents a viable strategy to treat disorders associated with dysfunction of these circuits. The phosphodiesterase (PDE) type 4 inhibitor rolipram has shown significant pro-cognitive effects in neurological disease models, both in rodents and primates. However, competitive non-isoform selective PDE4 inhibitors have a low therapeutic index which has stalled their clinical development. Here, we demonstrate the pro-cognitive effects of selective negative allosteric modulators (NAMs) of PDE4D, D159687 and D159797 in female Cynomolgous macaques, in the object retrieval detour task. The efficacy displayed by these NAMs in a primate cognitive task which engages the corticostriatal circuitry, together with their suitable pharmacokinetic properties and safety profiles, suggests that clinical development of these allosteric modulators should be considered for the treatment of a variety of brain disorders associated with cognitive decline.

## Introduction

Cyclic nucleotide signalling pathways play essential roles in synaptic plasticity and memory formation [1]–[5]. The modulation of, in particular, cAMP intracellular pools in neuronal cells are essential for the mechanism of action of many psychotropic molecules, and underlies the effects of numerous G-protein coupled receptor pathways [2], [6]. Phosphodiesterases (PDEs) play an important role in terminating cyclic nucleotide signalling via the hydrolysis of cyclic nucleotides [7]. There are twenty-one PDE proteins within eleven families (termed PDE1 – PDE11) with different expression patterns as well as varying affinities for cyclic nucleotides [5], [6], [8]–[11]. PDE4 inhibitors have, to date, been the most extensively investigated PDE inhibitors in the context of brain function and cognitive processes, and several studies highlight the role of cAMP and CREB signalling in neuroprotection and the modulation of neurogenesis in the dentate gyrus [12]–[15]. Deregulation of cyclic nucleotide and CREB signalling have been ascribed to several neurodegenerative conditions and cognitive impairment, including age-related memory loss, Alzheimer's disease (AD), and Huntington's disease (HD), amongst others.

We have a particular interest in the evaluation of PDE4 inhibitors for the treatment of Huntington's disease (HD). HD is a neurodegenerative, progressive, fatal autosomal dominant disorder characterized by loss and dysfunction of specific neuronal populations in the basal ganglia and in various cortical areas [16]–[20]. As a consequence, motor, psychiatric and cognitive deficits are characteristics of HD, presumably caused by dysfunctions in the cortico-basal ganglia circuitry affected in these patients. HD is caused exclusively by mutations in the *huntingtin* (*HTT*) gene. Although the exact nature of the interaction between huntingtin (HTT) protein and cyclic nucleotide signalling components is unclear, several reports point to dysregulation of cAMP pathways as a contributor to disease pathogenesis [21]–[30]. Loss of function of the transcriptional modulator CREB binding protein (CBP) has been postulated to contribute to the loss of neuronal function, as well as to the overall strong transcriptional deficits associated with HD, potentially through direct binding to HTT [21], [28], [31], [32].

PDE2, 9 and 10 enzymes have also been implicated in the control of brain functions relevant to Huntington's disease (HD) [3], [8], [9], [33]–[42]. Expression of PDE10 is downregulated in rodent models of the disease and in post mortem human tissues [43]. In the basal ganglia, cAMP elevation via both PDE4 and PDE10 inhibition modulates DARPP32 phosphorylation and influences behaviour during both movement and cognitive processes [4], [27], [40], [44]. In addition, the elevation of cAMP is required for memory processes in hippocampal synaptic plasticity and memory encoding and retrieval, which are also affected in HD rodent models [9], [12], [37], [39]–[41], [45]. Studies in rodents, non-human primates (NHPs), and humans with rolipram and other PDE4 active-site orthosteric inhibitors have highlighted the pro-cognitive effects in a variety of tasks involving both the hippocampal and the cortico-striatal systems [9], [40], [41], [45].

Rolipram, perhaps the most intensively studied active site PDE4 inhibitor, demonstrates preclinical efficacy in object recognition [46], water maze, and passive avoidance tasks in rodents [47], and in an object retrieval (OR) task (also called detour task) in the NHP [41]. Clinical studies using rolipram have investigated its potential utility in the treatment of affective disorders and for cognitive impairment, although its clinical development was halted due to adverse side effects including emesis and vasculitis in human and animal model studies [1], [48], [49], which have been observed at doses deemed too close to those necessary for efficacy. The narrow therapeutic index (TI) of rolipram prompted many investigators to pursue other strategies to separate the emetic liabilities from the beneficial effects. Such strategies have included the development of selective subtype PDE4D and PDE4B active site inhibitors [36], [38], [50]–[52], as well as the development of allosteric negative modulators (NAMs) of PDE4D [36]. In particular, a recent manuscript described the development of PDE4D NAMs with much improved TI over rolipram [36], [53], [54], due to their mechanism of inhibition (novel binding mode to the UCR2 domain of PDE4D7) and lack of full antagonism profile [36]. Based on a variety of tests conducted in *Suncus murinus*, dogs, and NHPs, these molecules were shown to have a much lower emetic potential, making them potentially useful agents for the treatment of cognitive and affective disorders. Therefore, we sought to explore whether PDE4D NAMs could affect cognitive performance relevant to HD.

Importantly, most PDE4 inhibitors in clinical development had a TI determined from studies conducted in multiple species. Typically the effective ‘dose’ for functional or cognitive improvement is extrapolated based on rodent tasks, whereas the adverse side effect dose is derived from toxicology studies conducted in larger species, such as dogs or NHPs. This may be misleading since in evolutionarily-divergent brain regions—such as the frontal cortex and basal ganglia—the modulation of cognitive processes in rodents versus primates (including humans) is likely to be divergent. Furthermore, the dose of psychoactive molecules might be dependent on the type and complexity of the cognitive task employed. We therefore assessed the effect of selective PDE4D NAMs in a task relevant to HD while monitoring for any adverse behaviour, including retching and emesis at the doses used during cognitive evaluation. We also monitored plasma and CSF levels of the drugs to define the exposure/efficacy relationship for potential clinical development.

The OR is a behavioral test designed to assess the functional and anatomical integrity of the frontostriatal pathway in NHPs and is adapted from an OR test used to cognitively assess children [55], [56]. This paradigm has been shown to involve executive function and its components including attention, response inhibition and planning, thought to involve the frontal lobe structures of the brain [55]. Performance in this task is impaired following lesions to the prefrontal and orbitofrontal cortices in the common marmoset [57], [58] and in the striatum in the capuchin monkey [59]. It has also been used to assess striatal dysfunction and cognitive impairment following SIV infection in macaques [60].

Pharmacological manipulations that elicit deficits in this task include dopaminergic depletions of the striatum in MPTP-treated monkeys [61], prefrontal serotonin depletion [62], and disruptions of this circuitry by subchronic administration of the NMDA receptor antagonist phencyclidine (PCP) [63], confirming the critical contribution of prefrontal corticostriatal circuitry. Antagonism of the D4 dopamine receptors reverse the subchronic PCP induced OR deficit [64]. Enhanced OR performance has been reported with acute dosing of the non-selective PDE4 inhibitor rolipram and with the PDE5 inhibitor sildenafil [41] in male Cynomolgus macaques (Macaca fascicularis). Other reports indicate the OR task is sensitive to nicotine, the acetylcholinesterase inhibitor donepezil [65], α7-nicotinic receptor agonists (such as GTS-21), and a GABA α5 inverse agonist [66]. Of note, elevations in cAMP signalling have been shown to impair performance in a spatial memory working task in aged (but not young) NHPs, so age is an important factor when assessing potential pro-cognitive effects of selective PDE inhibitors [67]. Therefore, the sex and age of the NHPs seem important to uncover potential cognitive effects in this task.

We evaluated both the pharmacokinetic characteristics and cognitive effects of two PDE4D NAMs, D159687 (dosed orally at 0.05, 0.5 and 5.0 mg/kg) and D159797 (dosed orally at 0.05, 0.5 and 1.0 mg/kg), and have monitored for adverse events over this dosing range. Female Cynomolgus macaques (4–6 year old) were used in this study. The two compounds display similar biochemical and cellular potencies (20–30 nM in a human cell-based assay using whole-blood [36]. D159687 and D159797 have high selectivity over other PDE enzymes, and good selectivity over the closely related enzyme PDE4B (20-fold selectivity; see [36]), setting them apart from rolipram; the latter served as a positive control in this cognitive paradigm at doses previously shown to elicit pro-cognitive effects during OR without inducing emesis (dosed at 0.003, 0.01 and 0.03 mg/kg intramuscularly) [41].

Our pharmacokinetic data indicate that the plasma clearance in NHPs of D159797 is ∼10-times lower than that of D159687 (0.17 vs. 1.65 L/h/kg, respectively). Since the compounds have similar volumes of distribution, the clearance difference results in a 10-fold difference in elimination half-lives (1.24 h for D159687and 10.4 h for D159797, respectively). Our studies replicated the pro-cognitive effects of rolipram in the OR task in young female NHPs, and demonstrate that a more robust pro-cognitive effect can be achieved with either of the PDE4D selective NAMs in this task, possibly because the dose ranging was not limited by the emergence of adverse events, as is the case with rolipram. The shorter half-life D159687 had an improved TI over D159797, which has a much longer half-life. These findings also demonstrate that selective modulation of PDE4D isoforms is sufficient for pro-cognitive benefit in this task.

## Materials and Methods

The present study adhered to the Association for Assessment and Accreditation of Laboratory Animal Care (AAALAC), the National Advisory Committee on Laboratory Animal Research (NACLAR) guidelines and was approved by the Institutional Animal Care and Use Committee of Maccine Pte Ltd (IACUC protocols #46-2007 amendment 27 and #162-2009). Animals were provided with environmental enrichment such as Kong toys, mirrors, rattles and foraging boards in addition to tactile (where possible), olfactory, auditory and visual contact with con-specifics. Prior to experimental investigations animals were habituated to study personnel via positive social interaction and to experimental techniques using positive reinforcement training to minimize stress and to mitigate any adverse effects on health, well-being and behavior. Study personnel remained consistent throughout the study and veterinary care was available around the clock. The in-house veterinarian examined each subject prior to study initiation and performed blood tests and physical examinations as required. There were at minimum twice daily observations of each subject for general health, alertness, and overall behavior; any abnormal changes were immediately reported to the attending veterinarian. Following dose administration animals were monitored every 10 minutes for a minimum of 2 hours. No animal was sacrificed during these studies; however emergency procedures and humane endpoints were outlined in the IACUC protocol.

### Subjects

Sixteen female, adult (4–6 years; weights 2.39–3.6 Kg) Cynomolgus macaques (*Macaca fascicularis*) were individually housed in an AAALAC accredited, GLP compliant facility with cage environmental enrichment (such as Kong toys, mirrors, rattles and forage material) in addition to tactile (where possible), olfactory, auditory and visual contact with con-specifics. Eight animals were assigned to the object retrieval study and eight to the pharmacokinetic (PK) evaluation group. Animals were trained for dose administration using positive reinforcement techniques to minimize stress and to mitigate any adverse effects on health, wellbeing and behavior. The air-conditioned colony room was maintained at 20±2°C, 50±20% humidity with a normal 12 h light-dark cycle (on 07:00, off 19:00). Food (Primate Lab Diet 5048, Purina Mills, USA) and water were available *ad libitum* and fresh fruit was offered twice daily. All experimental procedures were approved by the Institutional Animal Care and Use Committee of Maccine Pte Ltd (IACUC protocols #46-2007 amendment 27 and #162-2009) and were in accordance with AAALAC, NACLAR and good practice [68] guidelines.

### Cisterna-magna Cannulation Surgery

The eight animals assigned to the PK study were surgically prepared with indwelling cannulae inserted into the cisterna magna and connected to a subcutaneous access port to permit cerebrospinal fluid (CSF) sampling. Animals were sedated with ketamine (0.1 ml/kg of 100 mg/ml solution) and the dorsum of the head, neck, and scapular region shaved and prepared for sterile surgery. Isoflurane was delivered by mask to achieve a depth of anesthesia suitable for endotracheal intubation. Following intubation, anesthesia was maintained using isoflurane. Intraoperative monitoring included heart rate, oxygen saturation, end-tidal carbon dioxide, eye reflexes, jaw tone, body temperature (a water circulated heating pad was used to keep animals warm), and response to surgical stimulation.

Animals were placed in sternal recumbency with added padding under the trunk, allowing the head/neck to be manipulated into a flexed position. A final surgical scrub and site preparation was performed using iodine and a sterile drape applied around the surgical site. A longitudinal incision was made in the skin on the dorsal midline of the occipital region extending caudally. In the occipital region, the incision was extended through muscle layers and planes using sharp and blunt dissection, to the ligament/membrane overlying the cisterna magna between the occipitus and the dorsal process of the atlas. The sterile catheter and port was prepared to the correct length and filled with sterile saline. A small incision (∼1–1.5 mm) was made in the membrane and the catheter carefully inserted into the cisterna magna. The catheter was tunneled under the skin to the subcutaneous space between the occipital region and the rostral portion of the head.

Muscle and skin layers were sutured with absorbable suture. A suitable analgesic was administered during induction of anesthesia and twice daily during the 48 hours postoperatively. In addition, a suitable antibiotic was administered pre-operatively and for 5 days post-operatively. Animals were allowed two weeks to recover from surgery prior to any sampling and dosing being performed. During this time, the indwelling catheters were monitored and checked for patency and observation of clear and free flowing CSF.

### Compounds and Dose Formulation

Rolipram (0.3 mg/mL; Evotec, UK) was prepared fresh daily in a 10% Solutol in 90% saline (0.9% NaCl) vehicle. Rolipram formulations of 0.1 and 0.03 mg/mL were prepared from the stock 0.3 mg/mL stock solution via serial dilution. Rolipram was administered via the intramuscular route in the OR evaluation at a dose volume of 0.1 mL/kg in order to attain final dose levels of 0.03, 0.01 and 0.003 mg/kg respectively.

D159687 and D159797 (DeCode Genetics, Iceland), were supplied as the free base. Compounds had purity in excess of 97%. Dose formulations for the PK and OR study were prepared fresh daily and administered on the day of preparation. For the PK study intravenous (IV) infusions; D159687 (0.5 mg/mL) was formulated as a solution in 13% (w/v) PEG 400, 3% (w/v) Cremphor EL and 84% of 5% (w/v) Dextrose formulation. D159797 (0.5 mg/mL) was formulated in 15% (w/v) PEG 400, 1% (w/v) EtOH and 1% (w/v) Tween 80 in 0.9% NaCl vehicle. For PO administration (PK study and OR evaluation), D159687 and D159797 were prepared as solutions in a 0.5% (w/v) Poloxamer 188: 0.5% (w/v) HPMC: 0.4% Tween 80 vehicle at 5.0 mg/mL final concentration.

### Pharmacokinetics of D159687 and D15979

#### Animal Phase

Of the eight animals surgically prepared with indwelling cisterna-magna catheters, 6 animals were initially assigned to the PK study following catheter patency checks as described above. The animals were evenly assigned to groups 1 (D159687 pharmacokinetics) and 2 (D159797 pharmacokinetics) based on their body weight measured the day prior to dosing. These animals remained in that group for the duration of the study with the exception of one animal from group 1 which had to be replaced due to cistern-magna patency issues after IV dosing and prior to the oral phase of the study.

**Figure 1:**
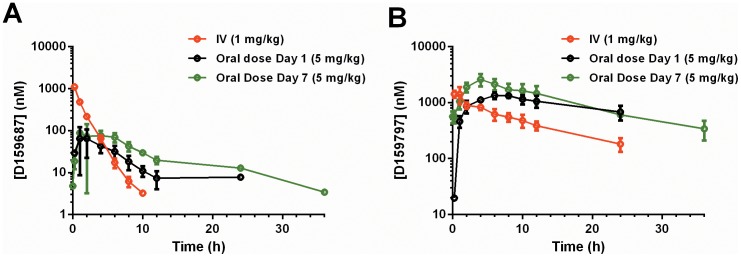
Pharmacokinetic analysis of PDE4D NAMs in female Cynomolgous monkeys. (A–B) Plasma exposure of D159687 (A), or D159797 (B) in female Cynomolgus monkey plasma following a single intravenous administration at 1.0 mg/kg, and on day 1 and day 7 after repeated daily oral administration at 5.0 mg/kg.

On Day 1 of the PK study, animals were administered 1 mg/kg D159687 (group 1, n = 3) or 1 mg/kg D159797 (group 2, n = 3) by intravenous infusion (Day 1). The animals then received a 9 day wash-out prior to the commencement of the determination of oral pharmacokinetic properties. In this instance, the animals were administered 5 mg/kg D159687 (group 1, n = 3) or 5 mg/kg D159797 (group 2, n = 3) by oral gavage daily for a period of seven days (study days 11–17). Dose volume for both D159687 and D159797 was 1.0 mL/kg. The results of this study are shown on Figure 1.

On Days 1 (IV), 11 (oral, day 1), and 17 (oral, day 7), whole blood (0.5 mL) was collected from the femoral vein of each animal. CSF (150 µL) was collected from the subcutaneous port in each animal using a sterile Posigrip ‘Huber point’ 22G x ¾ inch needle (Access Technologies, USA) at the same time as the blood collection. The area around the port was swabbed prior to insertion of the needle and 150 µL of CSF (dead volume) was removed immediately prior to the actual CSF sample collection. Blood and CSF were collected from each animal at pre-dose and following dose administration at 0.25, 1, 2, 4, 6, 8, 10, 12, and 24 h post dose on days 1 and 11 and additional blood and CSF sample was collected at 36h post-dose on day 17. All time points were timed from the start of dose administration. Whole blood was held on wet ice until centrifugation at 4°C (3500 rpm for 10 minutes) to allow plasma separation, after which two aliquots of plasma (at least 100 µL each) were directly transferred to individually labelled tubes and stored at −70±10°C until analysis. Two CSF aliquots of 50 µL were diluted with acetonitrile (1/1, by volume) and the remaining CSF aliquots (approximately 50 µL) were snap frozen in liquid nitrogen prior to storage −70±10°C until analysis.

#### Bioanalysis

Concentrations of D159687 and D159797 in dose formulation, NHP plasma and CSF were determined using an HPLC-tandem mass spectrometry (LC-MS/MS) method developed at Charles Rivers Laboratory (Preclinical Services, Shrewsbury). The matrix calibration standards were prepared at concentrations of 1.00–10000 ng eq./mL in Cynomolgus control plasma for plasma, and in 1∶1 by volume Cynomolgus CSF:acetonitrile for CSF. For dose formulation analysis, the calibration standards were prepared in 1∶1, by volume Mobile Phase A: Mobile Phase B at concentrations of 5.00–500 ng eq./mL. Diclofenac or reserpine were used as an internal standards (IS) as indicated below. The lower limits of assay quantitation (LLOQ) for D159687 were 2.73 nM in plasma, and 2.73 nM, 5.46 nM or 13.7 nM in CSF depending on the dilution of the sample. The lower limits of assay quantitation (LLOQ) for D159797 were 2.48 nM in plasma and 2.48 nM or 12.4 nM in CSF depending on the dilution of the sample. Results of the dose formulation analysis indicated that the measured concentrations of D159687 and D159797 were within 15% of the nominal concentration for both dose routes. Therefore, all PK estimations were based on target dose levels.

CSF samples were diluted in acetonitrile (1/1, by volume), at the time of collection. Dose formulation samples were diluted in a mixture of Mobile Phase A/Mobile Phase B (1/1, by volume), to bring them to a concentration within the range of the calibration standards. Analytes were extracted by protein precipitation (test tubes were kept on ice and protected from light), as follows: An aliquot (25 µL) of samples (plasma, diluted CSF or diluted plasma), matrix blanks, control blanks, and matrix calibration standards were placed into individual wells in 96-well plates. An aliquot (100 µL) of a solution containing diclofenac or reserpine as the internal standards (IS), (0.100 µg eq./mL in 0.1% formic acid in acetonitrile) was added to all samples except matrix blanks and solvent blanks. An aliquot (100 µL), of 0.1% formic acid in acetonitrile was added to matrix blanks. Samples were vortexed for 1-min and centrifuged for 5-min. Each supernatant was transferred to an individual well in a new 96-well plate. Immediately prior to LC-MS/MS analysis, an aliquot (50 µL) of Milli-Q water was added to each well, the plate was covered and vortexed for 1-min.

Analytes were separated by high performance liquid chromatography and quantified by mass spectrometry (LC-MS/MS). There were two mobile phases, Mobile Phase A consisted of 1.0% formic acid in water, and Mobile Phase B of 1.0% formic acid in acetonitrile. Two LC-MS/MS systems were used. The first one, used for the dosed analytes in plasma and dose formulation, consisted of an Agilent 1200 binary pump equipped with an Agilent G1367D autosampler and connected in tandem with an API5500 mass spectrometer. A Hypersil Gold (3µ, 20×2.1 mm) chromatography column at room temperature was used for analyte separation. The flow rate was maintained at 0.6 mL/min, and the gradient increased linearly from 5% to 98% Mobile Phase B in 1.2 min and held at 98% B for 0.5 min. The injection volumes were 2.5–10 µL. The second one, used for the dosed analytes in CSF and metabolites in CSF and plasma, consisted of a PE Series 200 micro pump equipped with a Leap CTC PAL autosampler and connected in tandem with an API4000 mass spectrometer. A Phenomenex Gemini (3µ, C18, 50×2.0 mm) chromatography column at room temperature was used for analyte separation. The flow rate was maintained at 0.3 mL/min, and the gradient increased linearly from 5% to 90% Mobile Phase B in 2 min and held at 90% B for 3 min. The injection volume was 30 µL.

In both instruments, the electrospray ionization source was set to scan in positive ionization mode, with a dwell time set to 100 ms. The multiple reaction monitoring (MRM) transitions were m/z 367.4 to 231.0 for D159687, m/z 403.4 to 217.0 for D159797, m/z 609.1 to 195.2 for reserpine (IS) and m/z 297.9 to 215.9 for diclofenac (IS).

#### Pharmacokinetic Calculations

Pharmacokinetic (PK) parameters were calculated by non-compartmental analysis using WinNonlin program, version 5.2 (Scientific Consulting Inc., Palo Alto, California). A model was selected based on the vascular (IV Infusion) or extravascular (oral gavage) routes of administration. For both routes, the pre-dose concentration was used as the concentration at time zero. Plasma and CSF concentrations below the limit of quantitation were treated as absent samples for the purpose of calculating the mean plasma concentration values or for calculating pharmacokinetic parameters.

The area under the plasma concentration versus time curve (AUC) was calculated using the linear trapezoidal method (linear interpolation). When appropriate, the terminal elimination phase of the PK profile was estimated using at least the last three observed concentration values. PK parameters describing the systemic exposure of analytes of interest in plasma or CSF were estimated from observed (rather than predicted) plasma concentration values, the dosing regimen, the AUC, and the terminal elimination phase rate constant (k_el_) for each group. The portion of the AUC from the last measurable concentration to infinity was estimated from the equation Ct/k_el_, where Ct represents the last measurable concentration. The extrapolated portion of the AUC was used for the determination of AUC_(0-inf)_. The percent bioavailability (%F) was calculated by dividing the dose normalized oral AUC_(0-inf)_ by the dose normalized iv AUC_(0-inf)_ times 100. The bioavailability calculations assumed concentrations were in the linear range.

### Behavioural Assessments - Object Retrieval (OR)

A total of n = 8 female *Cynomolgus* monkeys aged 4–6 years were trained in the OR task and were used for these studies, following a cross-over design. Animals were trained to ensure adequate and stable performance during the dosing studies. Animals were individually housed prior to the initiation of the study. Animals were habituated to the oral gavage method using positive reinforcement techniques for a minimum of 2 weeks prior to the initiation of OR testing to mitigate any adverse impact on behaviour. To ensure accurate dosing each individual was momentarily restrained in a ‘standing’ type primate chair to allow easy and gentle passage of the gavage tube. The oral doses were administered in a dose volume of 1.0 mL/Kg which was followed with 5 mL of purified water to ensure no residual compound formulation remained in the gavage tube. During dose habituation and the study the same study personnel administered the compounds and thus a non-stressful, familiar routine for the animals was achieved.

**Figure 2:**
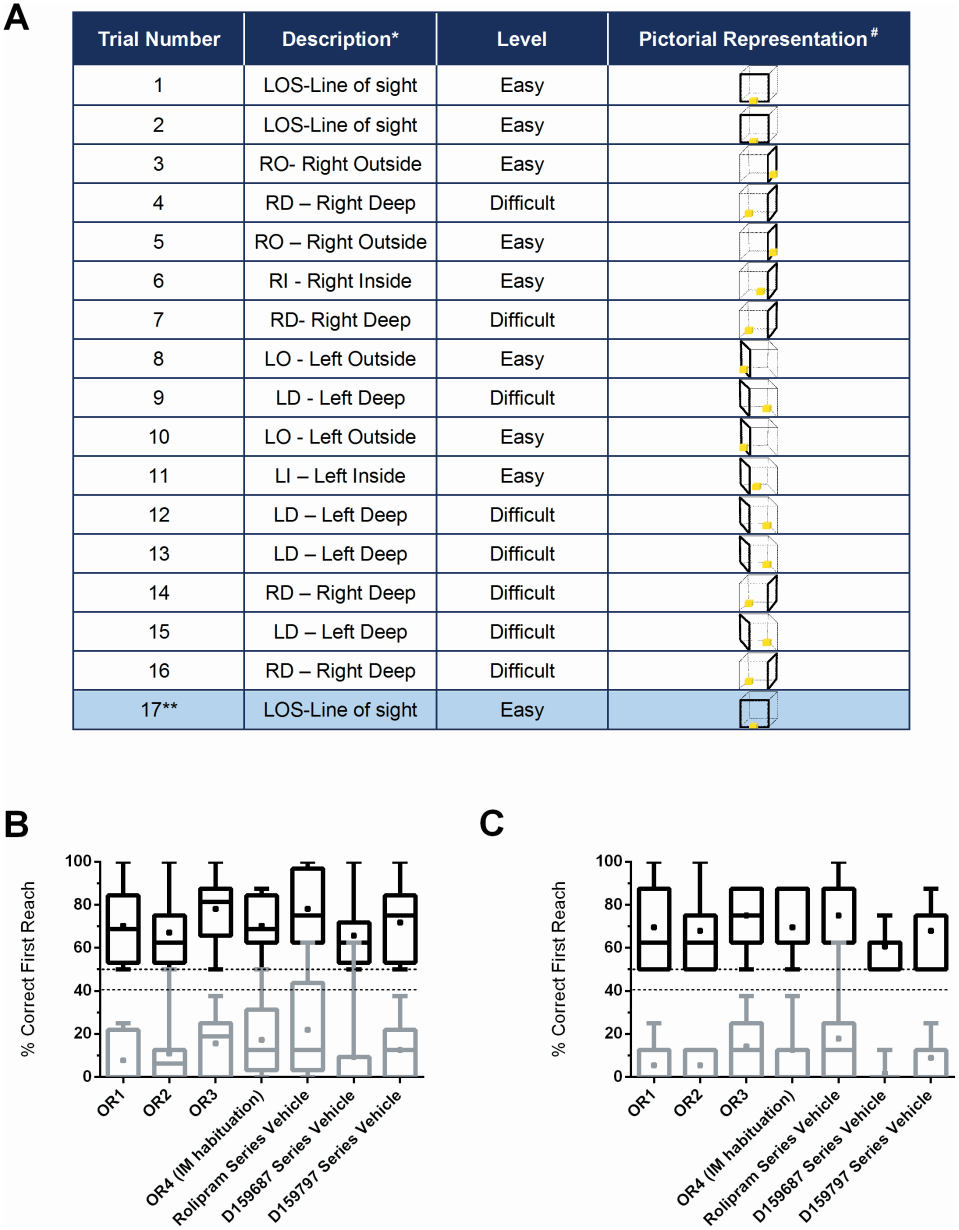
Object retrieval task schematic and baseline characterization. (A) Order of object retrieval task sessions for easy and difficult trials. Drawings illustrate the position of the reward in the boxes. (*) Left position will become right and right position will become left and so on in weekly rotation throughout the study. (#) The **bold** side of the cube represents the open side of the cube. (**) Trial 17 was for reward purposes and was not included in the data analysis; (B) Box (line indicates median, * indicates mean, box represents upper and lower 25 percentiles) and whisker (maximum to minimum) plots of all animal performance during the 4 training sessions and to vehicle administration during the testing phase on both easy (grey) and difficult (black) trials. Dashed lines indicate targeted performance – performance in easy trials >50% correct first reach, and performance in difficult trials <40% correct first reach. (C) Same data as in (B) but with high performer animal 3939 excluded. Criteria are largely met by exclusion of this animal. The outlier value during the rolipram vehicle trial is due to animal 7A5D.

The OR task [41], [55] requires the subject to retrieve a food reward from a clear acrylic box (dimensions  = 5×5×5 cm) with one open side which is positioned in front of the subject on a metal holder fixed to the outside of the home cage. The cube is presented to the subject with the open side facing left, right, or toward the monkey in a randomised set list of easy versus difficult trials. Food rewards (raisins or cubes of fruit pieces 1–2 cm^2^) were placed on the outer edge, inner edge, line of sight (all easy), or deep within the box (difficult). Each test session consisted of 17 trials [41], with an initial Easy phase of 3 easy trials followed by a Random phase of 9 trials (4 difficult and 5 easy, presented randomly for each session), followed by a Difficult phase of 4 difficult trials and finally one final Easy Trial (Figure 2A). The final easy trial was included for reward purposes and although the number of attempts was recorded the data were not used for analysis. For successive test sessions, the subject was presented with the trials in an order exactly the reverse of the previous session (previous left side exposure became right side exposure and vice-versa). Individual trials within one OR session were terminated if there were no reaches or successful retrieval of the reward within 2 minutes by the subject. The acrylic box was cleaned between trials to minimize cues which may influence subsequent task performance through easier identification of the cube entrance.

#### Training weeks and stable baseline performance evaluation

Animals were habituated to the metal frame prior to introducing the OR cube. During training, the animals were exposed to the OR trials as depicted in Figure 2A for 4 occasions (twice a week- OR trial # T1-T4 – data not shown). Importantly, during this period of training, all animals acquired the rewards even after an incorrect first reach. OR evaluations were then subsequently conducted once per week to discourage learning and thus ensure no performance shift during the pharmacology study (Figure 2B). Animals continued to be trained once per week for a period of 3 weeks immediately prior to pharmacology (OR1- 3; Figure 2B).

Baseline performance was targeted to be an average, stable performance of >50% correct responses in the easy tasks, with less than 40% correct responses in the difficult tasks (Figure 2B). All animals evaluated during this phase met these criteria by the end of the training session, with the exception of one animal, which was a baseline ‘high performer’ on difficult trials (Animal 3939). Figure 2C shows the stability of the baseline performance without inclusion of this particular animal. However, this animal was included in all subsequent dosing phases. For the subsequent evaluation of compounds on OR task performance, a within-subjects design was employed for this study randomised in a Latin-square design. OR behavioural scoring was completed in a blinded fashion. Testing was conducted as follows:

### Testing period (Weeks 1–16)

- Week 1: Animals were habituated to intramuscular injections daily using the ‘rolipram’ vehicle formulation (10% solutol (w/v), 90% saline vehicle in a 0.1 mL/Kg dose volume) Monday through Friday, and a single baseline OR trial evaluation was performed on Wednesday (OR4) (Figure 2A). Animals were dosed with vehicle 30 minutes prior to this behavioural evaluation.
- Weeks 2 to 5: Evaluation of rolipram in OR task. Following the stable baseline performance establishment (OR1 – 4), all animals were administered the 3 doses of rolipram (0.003, 0.01 and 0.03 mg/Kg) or vehicle intramuscularly 30 minutes before evaluation on the OR task, every Wednesday through Weeks 2–5 (OR5-8). All animals were dosed with vehicle on the preceding Monday and Tuesday to keep them habituated to the injections and avoid any Pavlovian conditioning to any pro-emetic effects of treatment, although no OR testing was carried out on those days. Treatment was administered at approximately the same time each day and administered via the intramuscular route at a volume of 0.1 mL/kg. Dosing was randomized per subject in a latin –square design.
- Week 6: Wash-out and habituation of the animals to handling and the oral gavage dosing procedure daily (Monday through Friday) was conducted. Animals were dosed with a 0.5% Poloxamer 188+0.5% HPMC +0.4% Tween 80 vehicle at a dose volume of 1.0 mL/Kg. This dose was washed down with approximately 5 mL of purified water. No behavioural testing was conducted during this ‘wash-out’ week.
- Weeks 7 to 10: The animals were given test compound D159687 every Wednesday over this 4 week period. All animals received the 3 dose levels of D159687 (0.05, 0.5 and 5.0 mg/Kg at a dose volume of 1.0 mL/Kg) or its vehicle using a Latin-square design so that all animals were tested in a randomized manner at each dose level. The test compound was administered 2 hours before evaluation on the OR task on each Wednesday (OR 9- 12). All animals were administered vehicle via oral gavage on Monday and Tuesday to keep them habituated to oral administration and to avoid any Pavlovian conditioning to any pro-emetic effects of treatment, although no testing was carried out on those days. Treatment was administered at approximately the same time each day. The doses were administered orally via gavage in a volume of 1 mL/kg and washed down with approximately 5 mL of purified water.
- Week 11: Maintenance of habituation to oral gavage/wash-out period. Animals were habituated to handling and the oral gavage dosing procedure daily (Monday through to Friday). Animals were dosed with a 0.5% Poloxamer 188+0.5% HPMC +0.4% Tween 80 vehicle at a dose volume of 1.0 mL/Kg. This dose was washed down with approximately 5 mL of purified water. No behavioural testing was conducted during this ‘wash-out’ week.
- Weeks 12 to 16: The animals were given test compound D159797 every Wednesday over this 4 week period. As in Week 7 to 10, animals were administered vehicle via oral gavage on Monday and Tuesday of each week to keep them habituated to oral administration and to avoid any Pavlovian conditioning to any pro-emetic effects of treatment. All animals intended to receive the 3 dose levels of D159797 (0.05, 0.5 and 1 mg/Kg) or its vehicle, randomized in a Latin-square design so that all animals were tested at each dose level (Weeks 12–15). The test compound was administered 2 hours before evaluation on the OR task on each Wednesday (OR 13–17). However, while the dose levels used were 0.05, 0.5 and 5.0 mg/Kg during week 12, the top dose level was lowered during week 13 to 1.5 mg/Kg and further reduced to 1.0 mg/Kg for weeks 13–16 following signs of retching at the highest dose (see results section). Thus the additional week was included in the dosing design (Week 16) which allowed all animals (except 7A5D) to receive a top dose of 1 mg/kg for evaluation. Deviations to the Latin square dosing design are depicted in Table S3.

#### Clinical Observations

Animals were closely monitored for the duration of the study, and were videotaped during task performance. Body weights were monitored weekly. Clinical observations were recorded prior to dose administration and after the completion of each OR task. Furthermore, a behavioural scoring record was used to record any changes in behaviour for each animal commencing at 5 minutes prior to dose administration and once every 10 minutes for up to 2 hours after dose administration.

#### Statistical Analysis

The mean percent correct first reaches, total reaches and barrier reaches for easy and difficult trials were analysed using repeat measures one-way analysis of variance (ANOVA), with dose group as the primary factor. Demonstrated observations of statistical significance were analysed via post-hoc Dunnett's analysis using the vehicle treatment as the reference control. Statistics are presented as F (DFn, DFd) and results were considered significant when p<0.05 and confidence intervals were set at 95%.

## Results

### Pharmacokinetics

Pharmacokinetic parameters for D159687 and D159797 are presented in Figure 1, Table 1 (D159687), and Table 2 (D159797).

**Table 1:** Summary of plasma pharmacokinetic parameters of D159687 following single intravenous administration at 1.0/kg, and on day 1 and day 7 after repeated daily oral administration at 5.0 mg/kg.

				
PK Parameters	Units	IV (1 mg/kg)	PO day 1 (5 mg/kg)	PO day 7 (5 mg/kg)
**AUC_(0-last)_**	nM.h	1649±68	447±162	869±198
**AUC_(0-inf)_**	nM.h	1657±69	477±178	920±222
**AUCNorm**	nM.h.kg.mg	1657±69	95±36	184±44
**Cl**	L/h/kg	1.65±0.07		
**Vdss (area)**	L/kg	2.10±0.38		
**MRT (area)**	h	1.27±0.17		
**Oral F**	%		6±2.8*	12.6±1.77*
**C_max_**	nM		80±36	121±73
**Tmax (obs)**	h		2.3±1.5	4.3±2.9
**t_1/2_**	h	1.24±0.17	3.1±0.7	4.3±1.9

Data presented as mean ± SD of 3 animals.

*Calculated from n = 2, as due to patency issues in catheter of one animal following IV dosing, one animal was replaced for po dosing, thus, it was not a cross over design.

**Table 2:** Summary of plasma pharmacokinetic parameters of D159797 following single intravenous administration at 1.0/kg, and on day 1 and day 7 after repeated daily oral administration at 5.0 mg/kg.

				
PK Parameters	Units	IV (1 mg/kg)	PO day 1 (5 mg/kg)	PO day 7 (5 mg/kg)
**AUC_(0-last)_**	nM.h	11918±2087	23019±4205	39720±7924
**AUC_(0-inf)_**	nM.h	14669±2909	42885±15527	47834±4493
**AUCNorm**	nM.h.kg.mg	14669±2909	8577± 3105	9567±899
**Cl**	L/h/kg	0.173±0.032		
**Vdss (area)**	L/kg	2.35±0.32		
**MRT (area)**	h	13.7±1.5		
**Oral F**	%		57.2±9.4	66.6±12.8
**C_max_**	nM		1400±155	2590±656
**Tmax (obs)**	h		7.3±1.2	4±0
**t_1/2_**	h	10.4±1.2	19.3±6.1	14.4±8.6

Data presented as mean ± SD of 3 animals.

#### D159687

Following IV administration of D159687, the plasma clearance was high (1.65 L/h/kg), the volume of distribution was moderate (2.1 L/kg) and the terminal half-life was 1.24 hr. (Table 1). After the first day of oral dosing, the oral bioavailability was low (6%) and increased by approximately 2-fold (13%) after the seventh single daily dose (Figure 1A and Table 1). Consistently, the oral AUC_0-last_ increased approximately 2-fold from 447 to 869 nM h. The oral elimination half-life also increased from 3.1 to 4.3 h with multiple days of dosing. The T_max_ was quite variable between animals (2.3±1.5 h and 4.3±2.9 h at Days 1 and 7, respectively), with mean C_max_ values of 80±36 nM and 121±73 nM at Days 1 and 7, respectively. Consistent with the short terminal elimination half-life (IV), the AUC_0-24_ were similar to the AUC_0-inf_, indicating that in the absence of a change in absorption and/or clearance mechanism, D159687 will not accumulate. The low oral bioavailability was attributed to significant first pass metabolism. This conclusion was supported by the expected *O*-dealkylation of the compound (not shown) and was consistent with the high plasma clearance. However, contributions of dissolution limited absorption to the low oral bioavailability cannot be ruled out. The higher oral bioavailability and corresponding AUC and C_max_, together with the approximately 2-fold increase in elimination half-life observed on day 7, after multiple oral doses, could be a consequence of a change in the clearance mechanism(s), or enhanced absorption. Given the moderate volume of distribution of the compound, it is unlikely that the increase in AUC and half-life are an artifact of an inadequate bioanalysis limit of quantitation for a drug of a bi-phasic elimination profile. While these observations suggest the possibility that D159687 is either a time-dependent inhibitor of a monkey CYP activity, or an inhibitor of an active efflux mechanism, neither of these options are favored, as the exposure of the known metabolites increased with multiple days of dosing (data not shown), and inhibition of an efflux transporter would have resulted in a more prolonged half-life. Thus, we attribute the increase in D159687 AUC with days of dosing to a moderate increase in absorption.

#### D159797

D159797 had a much slower clearance (0.17 L/hr/kg), with a similar volume of distribution at steady state (2.35 L/kg), and a much longer elimination half-life (13.7 hr), than D159687 (Figure 2A and Table 2). The compound had an improved oral bioavailability (57% and 66% on Day 1 and Day 7). Plasma C_max_ was higher than for D159687, with mean values of 1400±155 nM on Day 1 and 2590±656 nM on Day 7 (Table 2). The oral AUC_0-24_ increased with days of dosing as driven by the time that it took to achieve steady state concentrations with the long elimination half-life (Table 2). Given the different clearance rates and oral bioavailability of these two compounds, we tested both molecules in the OR task and monitored for potential adverse effects.

Compound exposure in CSF was measured to assess if it could be used as a surrogate for assessment of unbound compound concentration in CNS, as for most drugs examined there has shown to be a reasonable correlation (within ∼3-fold)[69]. However, neither D159687 nor its known metabolites (data not shown), or D159797, were detected to any significant degree in the CSF after either IV or PO dosing (Table S1 and S2). In order to assess whether the compounds could potentially suffer from active CNS efflux, which is one mechanism that could account for the low detection in primate CSF, we conducted *in vitro* MDR1-MDCK assays with D159687 as an exemplar. These experiments showed D159687 to be low-to-moderately permeable (P_app_ A-B 44 nm/s) with an efflux ratio (ER) of 0.9, demonstrating that D159687 does not act as a MDR1/Pgp substrate and active efflux from the CNS is unlikely to occur via this mechanism. This finding is consistent with the *in vivo* PK data reported by Burgin et al. [36], who showed that the total brain: plasma AUC ratio for D159687 was ≥1 across rodents and primates.

Another potential reason why either D159687 or D159797 were not detected to any significant extent in the CSF could be due to high protein binding (both to plasma and/or brain tissue) which would limit the free (unbound) brain concentration of drug. The plasma protein binding of D159687 was measured in monkey plasma *in vitro* using equilibrium dialysis to determine if this was a significant issue. Binding was independent of incubated concentration (at 1, 5 and 10 µM) and showed that D159867 was moderate-to-highly plasma protein bound (F_u_ 0.07). Adjustment of the total plasma AUC by F_u_ to estimate unbound plasma AUC (AUC_uu_) following oral dosing gave adjusted values of between 33 - 61 nM.h at day 1 and day 7, within the effective range for target inhibition (IC_50_ of 28 nM).[36] Given that it is generally accepted that *in vitro* protein binding is a notoriously poor predictor of the central efficacy of orally administered drugs, since unbound drug concentration at the site of action is controlled by highly dynamic simultaneous physiological actions *in vivo* (ie permeation, target binding, metabolism, transport and movement between tissue and cellular compartments) [70], [71], our results using CSF sampling cannot confirm or rule out central target engagement at the doses we tested, suggesting that CSF sampling for the PDE4D NAMs lacks the sensitivity to be used as a biomarker of free brain drug concentration, at least at the limit of detection we achieved in the CSF matrix (LOQ 10-20 nM for most samples). Thus an alternative translational endpoint would be recommended to estimate target engagement in a clinical setting.

### Pro-cognitive Effects of Rolipram and selective PDE4D Negative Allosteric Modulators Evaluated via the Object Retrieval (OR) Task

The OR paradigm employed here is shown in Figure 2A. In this paradigm, we employed a cross-over randomized testing design, with weekly drug wash-out periods between the different drugs tested. A total of 8 female *Cynomolgus* monkeys (4–6 years of age) were trained with the aim to reach a stable baseline in the easy tasks, while performing consistently at less than 40% correct first reach in the difficult tasks (OR 1–4; Figure 2B). This level of performance would allow us to assess whether compounds would exhibit a pro-cognitive effect, as judged by any subsequent enhancement of the monkey's performance in the difficult trials during the dosing test periods. A consistent high level of accuracy in the easy trials (>50%) would also allow for detecting any cognitive impairment produced by drug administration. All animals selected, with the exclusion of one, met these criteria. The outlier animal, “3939”, was noted to be a ‘high performer’ in the difficult tasks at baseline. Figure 2C shows the performance of animals over the baseline period with the exclusion of this particular animal. Additionally, a post-hoc analysis of the performance of all animals after dosing with vehicle after completion of the study (Weeks 2–16; vehicle dosing during the Rolipram trial (OR trial # 5–8, IM), D159687 (OR trial # 9–12; PO) and D159797 (OR trial # 13–17; PO), also showed that the animals did not reveal any significant shift in performance on either the mean percent correct first reach for easy trials (F[3.73, 26.09] = 0.914, p = 0.47) or difficult trials (F[3.011,21.1] = 1.2, p = 0.33; see Figure 2B). In summary, our data suggest that we can accurately evaluate drug effects without confounds in the interpretation of vehicle, route of administration, study design length (repetitive trials) or individual animal performance at baseline.

#### Rolipram

After establishing baseline performance, we next tested the efficacy of the PDE4 inhibitor rolipram, dosed at 0.003, 0.01 and 0.03 mg/kg intramuscularly (IM). This compound was chosen as a reference compound, as it was previously reported to show efficacy in this task at 0.01 and 0.03 mg/kg tested via IM administration [41]. Higher doses of rolipram inevitably induce emesis: in a separate study, a dose of 0.05 mg/kg IM resulted in all animals retching (Maccine, personal communication); emesis at 0.06 mg/kg was noted in Burgin *et al* [36], and in Rutten *et al*, at 0.1 mg/kg, emesis was noted in 12 of 14 monkeys.[41] For these reasons, and with the TI of rolipram already firmly established by these studies, we limited our maximum dose of rolipram in the cognitive paradigm.

**Figure 3:**
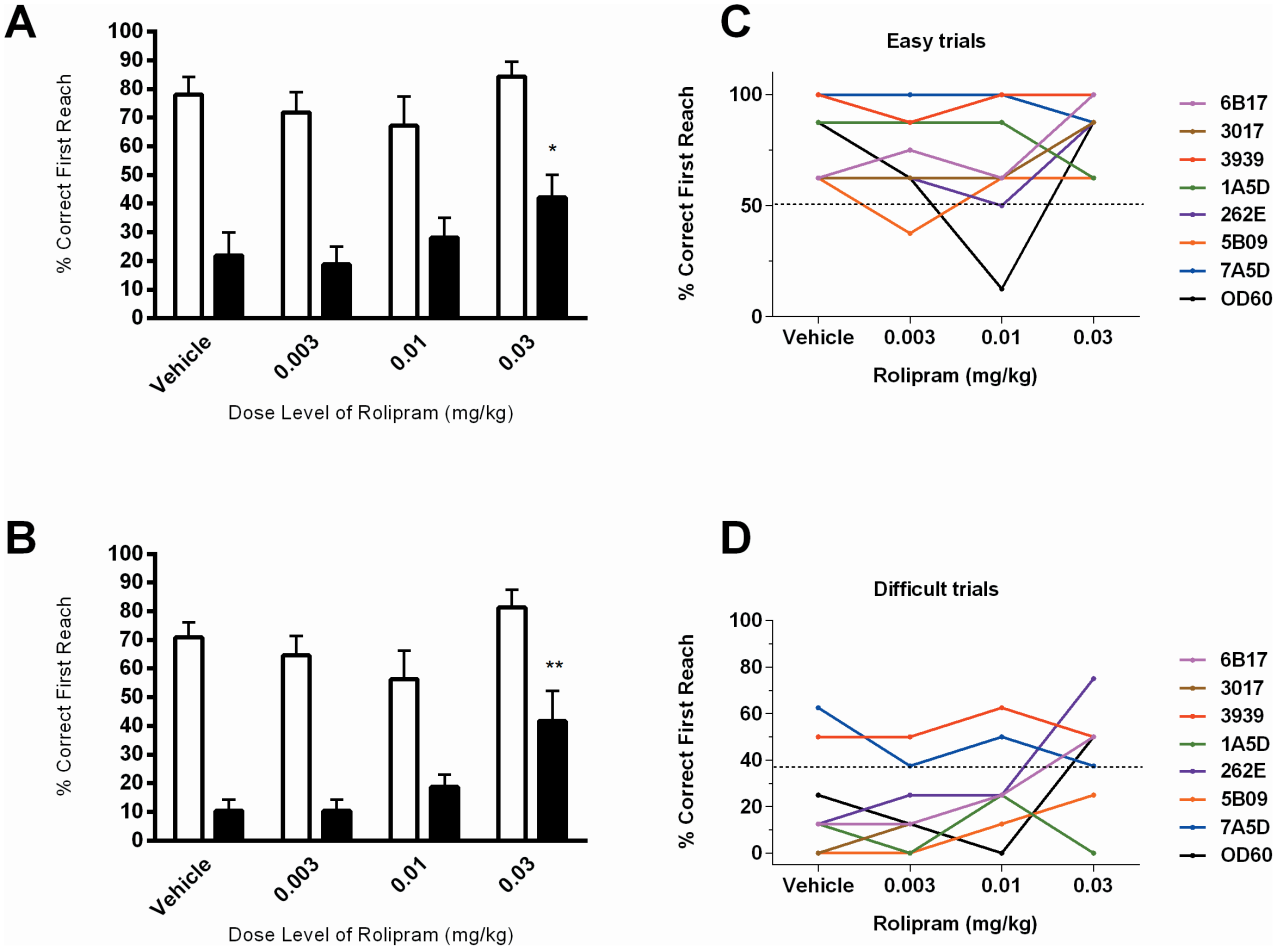
Influence of the PDE4 inhibitor Rolipram on the percent correct first reaches for both ‘easy’ and ‘difficult’ level OR performance. (A–B) Effects of rolipram on easy (open bars) versus difficult tasks (black bars) (A), and after exclusion of ‘high baseline performing animals’ 3939 and 7A5D (B). Values are shown as mean ± SEM. Asterisks denote significant differences from vehicle treatment (*p<0.05, **p<0.01) following repeat measures one-way ANOVA and Dunnett's post-hoc analysis. Individual animal performance plot in the easy trials is shown in (C) and in the difficult trials is shown in (D).

No significant improvement in the mean percent correct first reach for easy level trials was observed following rolipram administration (F [3], [21] = 1.641; p = 0.21), (Figure 3A and B). A significant improved OR performance was observed on rolipram dosing as determined by the mean percent correct first reach on the difficult trials (F [3], [21] = 3.789; p = 0.026); (Figure 3A and D). Post-hoc Dunnett's comparison revealed a significant increase (p<0.05) in the mean percent correct first reaches during difficult trials at the highest dose level of rolipram (0.03 mg/Kg; 42±8%) when compared to vehicle treatment (22±8%). Figure 3D shows the individual animal performance in the difficult trials. Note that the previously identified ‘high performing’ animal, animal 3939, and one additional animal, 7A5D, who performed better than during the training period, showed no obvious improvement on rolipram dosing. Removal of these animals from the group mean (Figure 3C) offered some improved significance on dose effect with difficult trial performance (F [3, 15 = 6.745] p = 0.004), with a significant effect in the mean percent correct first reaches during difficult trials at the highest dose level of rolipram (0.03 mg/Kg; p<0.01). No significant change in the number of barrier reaches or total reaches were observed for either easy or difficult level trials following rolipram administration (data not shown).

Animal 5B09 was the only animal that showed adverse reactions, in response to the highest dose (0.03 mg/kg) of rolipram tested. Retching and severe salivation was seen 20 minutes post dose. Salivation became moderate 30 minutes post dose and further decreased to mild salivation by 40 minutes post dose. This is in line with previous observations underscoring the narrow therapeutic index of rolipram.

#### D159687

Following a 1 week wash out and oral gavage dosing habituation, we subsequently tested OR performance of the same animals following D159687 administration (0.05, 0.5 and 5.0 mg/Kg by PO gavage). All animals completed the dosing as designed. However, animal 7A5D showed some ‘potential’ adverse effect to dosing at the highest dose level of 5 mg/kg. While no signs of retching or emesis were observed, at 100 to 120 minutes post-dose, 7A5D was observed to be hunched up in the corner of her cage with her head down and her hands interlocked behind her head. She was responsive to interaction and would eat ‘free’ raisins, but was not interested in doing the OR task and did not complete the task at this dose level. However, since this observation and in subsequent unrelated studies, this animal has been frequently observed to display such behaviour, and thus these effects cannot be clearly attributed to compound effects (Maccine, personal communication).

**Figure 4:**
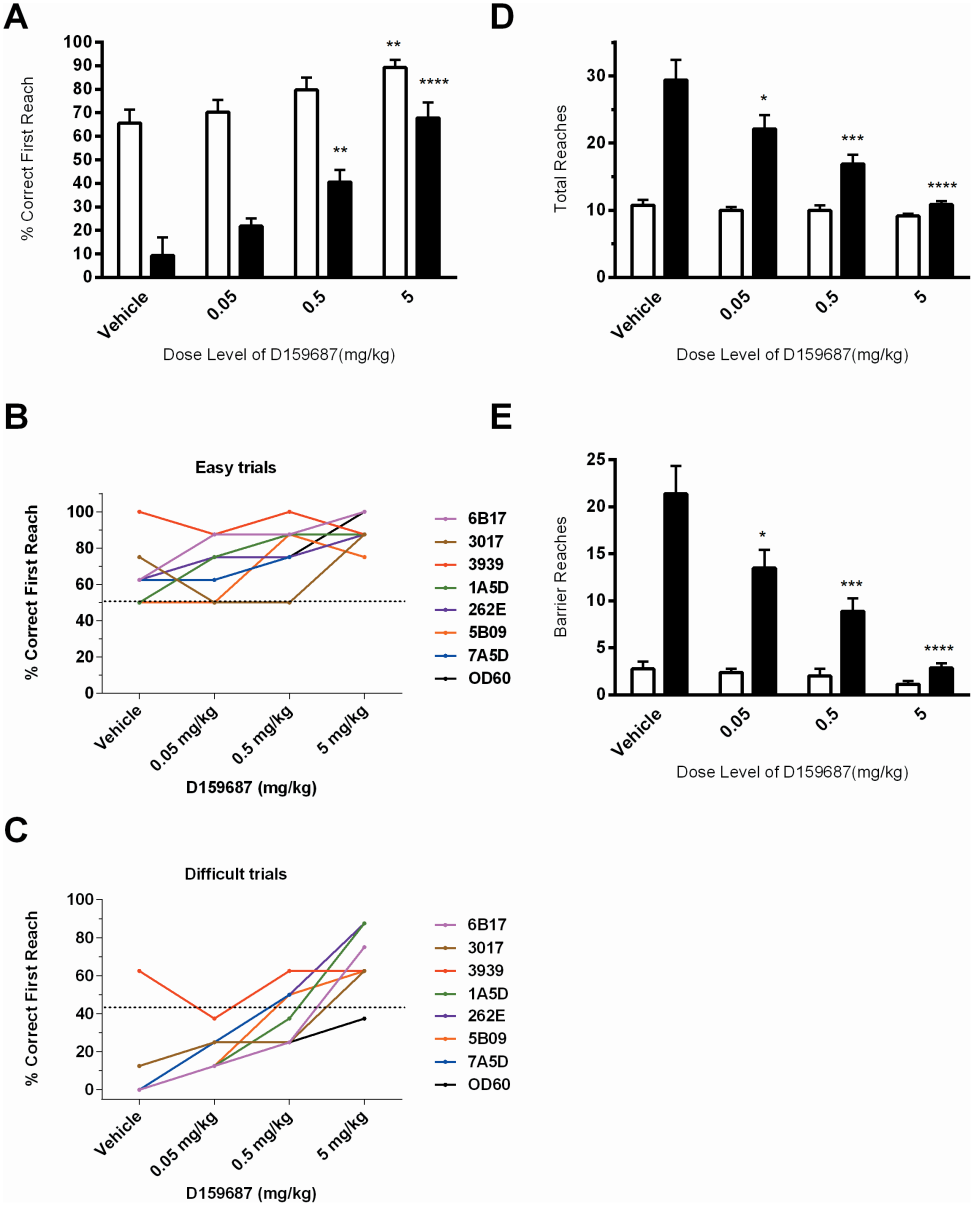
Influence of D159687 on OR trial performance. Effects of D159687 on easy (open bars) versus difficult task (black bars). Dose-dependent improvement in (A) mean percent correct first reach on difficult task performance, with modest improvement on easy trial performance. Individual animal performance plot in easy trials (B) and in difficult trials (C). (D–E) Dose-dependent reduction in the total number of reaches (D) and barrier reaches (E) on difficult taks. (A, D, E) Values are listed as mean ± SEM (n = 8 for vehicle, low and mid-dose groups, n = 7 for high dose group). Asterisks denote significant differences from vehicle treatment (* p<0.05, **p<0.01 and ***p<0.001) following repeat measures one-way ANOVA and Dunnett's post-hoc analysis (n = 7, due to non-completer 7A5D).

Administration of D159687 demonstrated a significant, dose-dependent increase in the mean percent correct first reach for difficult trials when dose group was considered a factor (F[3], [18] = 19.25; p<0.0001; as well as a lesser but significant increase in the mean percent correct first reach for easy level trials (F[3], [18] = 4.667; p = 0.014), when including all animals in the analysis that completed the task in full (n = 7; Figure 4A). Post-hoc Dunnett's comparisons revealed a significant increase in the mean percent correct first reaches during the easy trial at 5 mg/kg (p<0.05), and in difficult trials at both the 0.5 mg/Kg (p<0.01) and 5.0 mg/Kg (p<0.0001) dose level of D159687 (39±6% and 68±7% respectively) when compared to vehicle treatment (11±9%). Figure 4D and 4E show the individual animal performance (n = 8). Note that in the difficult trials, all animals with the exception of the ‘high performer’ 3939 appeared responsive to drug and improved their performance on D159687 administration. Administration of D159687 also demonstrated a significant, dose dependent decrease in the observed total number of reaches during difficult trials (F[3], [18] = 16.62; p = <0.0001), but not easy trials (F[3], [18] = 1.04; p = 0.40; Figure 4B). The number of barrier reaches were also significantly reduced during only the difficult trials (F[3], [18] = 17.11; p = <0.0001) when dose group was considered a factor (Figure 4C). Post-hoc Dunnett's comparisons revealed a significant decrease in both the total number and barrier reaches during difficult trials at each dose level of D159687 when compared to vehicle treatment (see Figure 4D and E for significance). The results were quantitatively much clearer than those observed with rolipram.

#### D159797

**Figure 5:**
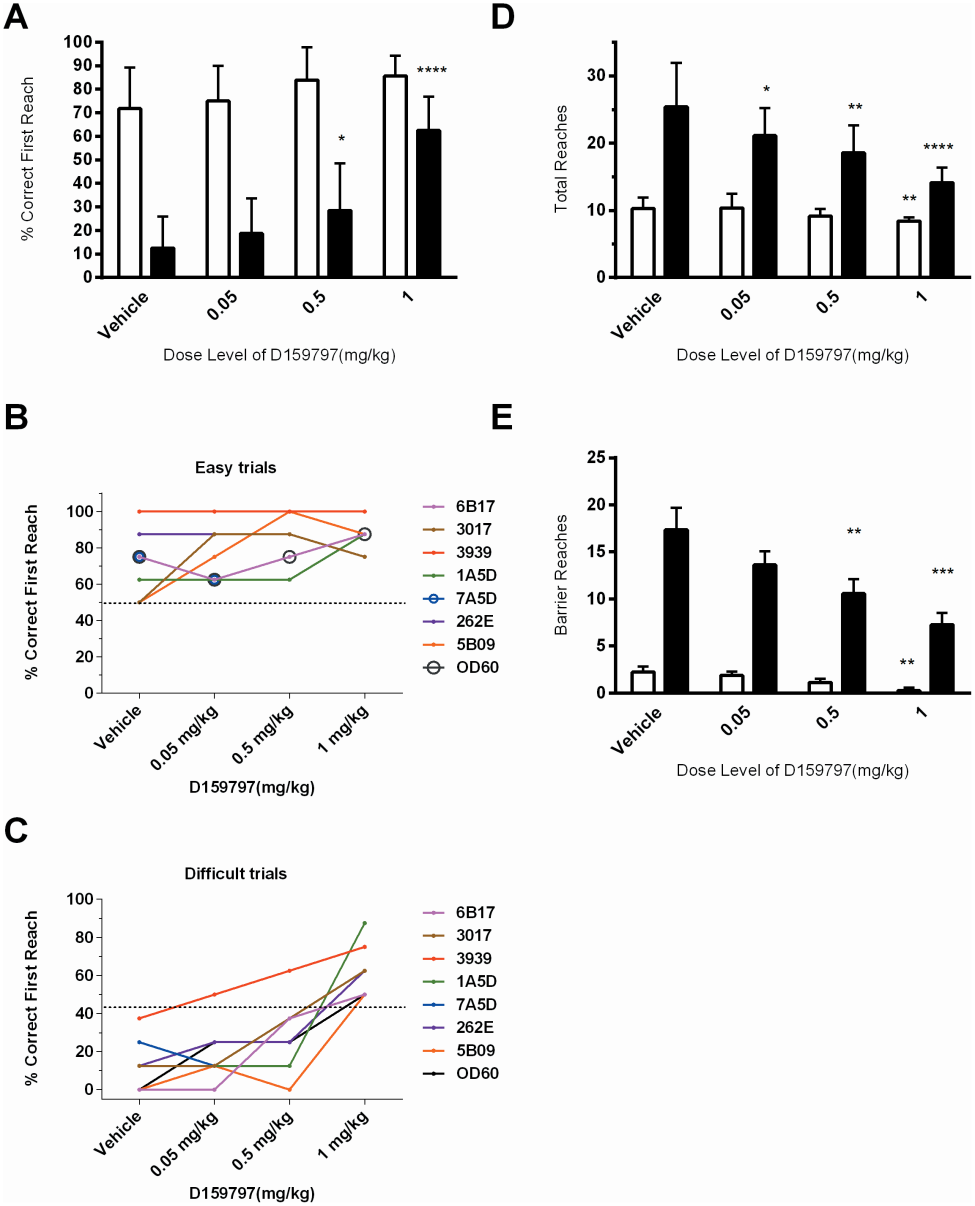
Influence of D159797 on OR trial performance. Effects of D159797 on easy (open bars) versus difficult tasks (black bars). Dose-dependent improvement in (A) mean percent correct first reach on difficult task performance. Individual animal performance plot in easy trials (B) and in difficult trials (C). (D–E) Dose-dependent reduction in the total number of reaches (D) and barrier reaches (E) on difficult tasks. (A, D, E) Values are listed as mean ± SEM (n = 8 for vehicle and low dose, and n = 7 for mid and high dose group). Asterisks denote significant differences from vehicle treatment (* p<0.05, **p<0.01 and ***p<0.001) following repeat measures one-way ANOVA and Dunnett's post-hoc analysis (n = 7, due to non-completer 7A5D).

Following a further 1 week wash out and maintained oral gavage dosing habituation, we tested OR performance of the same animals following D159797 oral administration. The experimental dosing paradigm had been set up to originally test D159797 at equivalent doses to D159687 (0.05, 0.5 and 5.0 mg/Kg by PO gavage) using a randomized Latin-square design. We did not anticipate problems with these doses following the findings that no adverse events were seen at the highest dose during the PK evaluation, where (different) animals were dosed for seven consecutive days without any adverse events. However, on the first week of dosing (Week 12), emesis was observed in the two subjects randomised to receive the top 5 mg/Kg dose (262E and 3017). Animal 262E did not attempt the OR task, and at 2 hours post-dose was retching and salivating severely, which persisted in a milder form to 5 hours post-dose, followed by a full recovery. Animal 3017 did complete the OR task, but was observed to retch, vomit and hyper-salivate on immediate completion of the task, but then behaved normally for the rest of the observation session (5 hr. post-dose). As a result, the top dose level was subsequently lowered to 1.5 mg/Kg on Week 13. One animal received this dose (6B17), however following the successful completion of the OR task, mild to moderate retching was also observed in this subject, which persisted intermittently for the 5 hr. post-dose observation period. Due to these clinical observations the dose levels were adjusted to a maximum dose level of 1.0 mg/Kg for D159797 for subsequent weeks. No adverse clinical observations were noted throughout the rest of the treatment. In order to make sure that all animals received the readjusted top dose of 1 mg/kg, an additional week was added to the protocol design. The dosing protocol is depicted in Table S3. However, one animal (7A5D) failed to complete the OR task at 0.5 mg/kg. In this instance, no hyper-salivation, retching or emesis was observed and the animal would take ‘free’ food rewards. Notably, this was the same animal that had failed to perform the task at 5 mg/kg in the previous D159687 testing period, and had, in subsequent unrelated studies, shown to exhibit the same disinterested behaviour in task performance (Maccine, personal communication). This animal was excluded from the 1 mg/kg evaluation, but we cannot ascribe the lack of performance when dosed at 0.5 mg/kg with confidence to any adverse effect of the drug at this dose level. As a result, all data presented in Figure 5 graph all data (n = 8 for vehicle, low and mid dose groups, with n = 7 for the 1 mg/kg group), but repeat measures ANOVA statistics described exclude subject 7A5D (n = 7 throughout study for all completers).

There was no significant increase in the mean percent correct first reach for easy level trials following D159797 administration (F[3], [18] = 2.11; p = 0.13), but a significant increase in mean percent correct first reach for difficult level trials was observed (F[3], [18] = 28.21 p<0.0001). Post-hoc Dunnett's comparisons revealed a significant increase in the mean percent correct first reaches during difficult trials at the 0.5 mg/kg (29±8%, p<0.05) and 1.0 mg/Kg (63±6%, p<0.0001) dose level of D159797 when compared to vehicle treatment (11±5%; Figure 5A). Notably and similarly to D159687 observations, in the difficult trials, all animals that completed the task showed an enhanced performance in a dose-dependent manner, and at the highest dose, every animal performed better than under vehicle conditions. Administration of D159797 also demonstrated a significant, dose dependent decrease in the observed total number of reaches during difficult trials (F[3], [18] = 14.29; p = <0.0001), and in easy trials (F[3], [18] = 4.543; p = 0.015; Figure 5D). The number of barrier reaches were also significantly reduced during in both easy (F[3], [18] 5.04, p = 0.01) and in the difficult trials (F[3], [18] = 10.62; p = 0.0003) when dose group was considered a factor (Figure 5E). Post-hoc Dunnett's comparisons revealed a significant decrease in the total number of reaches at each dose level of D159797, and a significant decrease in the number of barrier reaches at 0.5 and 1 mg/kg during difficult trials when compared to vehicle treatment (see Figure 5D and E for significance).

## Discussion

We demonstrate here a robust pro-cognitive effect of two selective PDE4D NAMs in young, sexually mature female NHPs in a task that is sensitive to frontal corticostriatal function. In addition, we replicate previous findings that rolipram also exerts such pro-cognitive effects in this task; although with reduced efficacy compared to the selective PDE4D NAMs. This could, as previously suggested, be a consequence of the different binding mode for the allosteric versus orthosteric ligands [36], or possibly to the potential contribution of PDE4B to the adverse side-effect profile of rolipram [36], [52]–[54], [72], which resulted in us capping the maximum dose we used in this study, based on the well-established emetic liability noted in several studies at doses greater than 0.05 mg/kg. [36], [41] It is important to note, that in our study, versus the study conducted by Rutten *et al.* [41], we only used female monkeys 4–6 years of age. Differences in the magnitude of the pro-cognitive effects of rolipram (our positive control) seen in the previous study, and our study, must be interpreted with this in mind. The current data obtained in female animals illustrates the same effect size at the 0.03 mg/kg dose of rolipram as that observed by Rutten *et al.*, although significance was not observed at 0.01 mg/kg. Indeed, gender differences may be a contributing factor to the dose response profile differences between the 2 studies. Alternatively, this difference may simply reflect different group sizes (n = 12 males in the Rutten publication [41] and n = 6 in the current manuscript) or age ranges (Rutten *et al* studied males aged 5–12 years whilst we studied a tighter age range in females of 4–6 years of age). A direct and suitably controlled gender/age study would be required to investigate these differences and their effect on the efficacy of these molecules.

The maximal effects of both D159687 and D159797 in the OR task were most pronounced in the difficult trials, where a potential recruitment or enhancement of synaptic function can be observed with increased task difficulty. Given that there is currently no primate genetic model of HD, task complexity in striatal-dependent behavioural tasks might serve as an alternative way to identify therapeutic compounds with cognitive-enhancing effects in disorders of the basal ganglia, such as HD, attention-deficit hyperactivity disorder (ADHD), autism or schizophrenia. Our data, in addition to the well documented pro-cognitive effects of PDE4 inhibitors in hippocampal-dependent tasks, argues for the development of PDE4 modulators with improved safety margins for a wide range of mental disorders.

For D159687, the minimum effective dose (MED) in the OR trial was 0.5 mg/kg against the primary endpoint measure of improvement in mean correct percent first reaches in difficult trials, and in which we also saw highly significant and effective reduction in total and barrier reaches during difficult trial performance. However, it should be noted that at 0.05 mg/kg PO there was a significant and effective reduction in both total reaches and barrier reaches during difficult task performance, but there was no significant improvement in mean percent correct first reaches (p = 0.14). Furthermore, we observed no evidence of adverse effects in female NHPs at doses up to 5 mg/kg in either the OR trial, where best dose effect was observed, or in the PK trial with seven day consecutive dosing, where AUC_(0-last)_ and C_max_ were 869±198 nM.h and 121±73 nM, respectively, giving an estimated AUC(_0-last)uu_ and C_max uu_ of ∼61 nM.h and 8.5 nM respectively (assuming plasma F_u_ = 0.07).

From published data [36] we know that D159687 elicits emesis at 30 mg/kg but not 10 mg/kg in NHPs, suggesting the “no adverse event limit” (NoAEL) may lie somewhere between these two doses. Taking the maximum NoAEL observed as 10 mg/kg, the therapeutic index over MED can conservatively be *estimated* as likely in excess of 20-fold (10 mg/kg/0.5 mg/kg), with the therapeutic index above best dose ∼2 fold. Although we make this estimate on a dose per dose basis between our study and that of the reports of NoAEL in NHP,[36] importantly both studies use exactly the same method of administration (oral gavage), in the same formulation, suggesting that exposure would not have greatly differed between the two studies. While we believe this is a valid estimate, a dedicated within subject design to assess MED/best dose for cognitive effects versus emetic threshold dose would be needed to provide a precise TI.

For D159797, the minimum effective dose in the OR trial was also 0.5 mg/kg against all measures evaluated during difficult trial performance. Similar to D159687, beneficial activity at 0.05 mg/kg PO was apparent. There was a significant reduction in total reaches with a trend to reduction in barrier reaches during difficult task performance, but there was no significant improvement in mean percent correct first reaches (p = 0.17). At the highest dose of 1 mg/kg, the drug response was highly significant and effective in all the parameters evaluated in our hands, representing the ‘best dose’ level. However, conversely to D159687, emesis/retching was observed with D159797 at 1.5 mg/kg (n = 1 of 1) and 5 mg/Kg po (n = 2 of 2) during the OR trials. This was somewhat surprising to us as no signs of emesis were observed in animals tested for 7 consecutive days at 5 mg/kg during the PK evaluation for this compound (n = 0 of 3). For D159797, a dose of 5 mg/kg corresponded to a plasma exposure AUC_(0-last)_ and C_max_ of 39720±7924 nM.hr and 518±131.2 nM, respectively. However, the emergence of clear adverse events with D159797 at doses >1.5 mg/kg), but not with D159687 at 5 mg/kg, suggests that there was no obvious tolerance to the effect of the PDE4D NAMs due to repeated drug administration, as D159797 was tested last in sequence. While this could have confounded the interpretation of our study if the results had been different, the choice to test D159797 last in the OR trial was deliberately chosen, based on the longer half-life of this compound, and with no *a priori* knowledge of whether long term effects would be anticipated. The extremely clear dose-dependent effects observed with the PDE4D NAMs when the dosing level was randomized in the within-subject design for each of the PDE4D NAMs tested also underscores that neither tolerance, or conversely, long-lasting effects of the compounds outliving the designated 1 week washout period, was a factor.

What pharmacokinetic parameter drives efficacy versus emesis in the PDE4 NAMs? A comparison of exposures at the reported adverse effect limit (AEL) using estimated AUCs (assuming scaling linearity) between D159687 (30 mg/kg; [36]; AUC_(0-last)_ and C_max_ estimated to be ∼5215 nM×hr and 726 nM) and D159797 (1.5 mg/kg; estimated AUC_(0-last)_ of ∼11,916 nM*h and C_max_ of 155 nM) would estimate that the AUC for D159797 is approximately 2.3-fold higher when compared to D159687. However, the C_max_ was estimated to be lower for D159797 at the AEL (726 nM for D159687 vs 155 nM) suggesting that emetic effects could be driven primarily by AUC. Additional evaluation of this class of compounds with differing exposures and clearance rates will be needed to clarify this, along with a more formal tolerability trial at higher doses to obtain a precise AEL for each compound.

In summary, our data and that obtained in rodent cognitive testing [36] suggests that this class of molecules exhibits robust cognitive effects at seemingly very low exposures. We attempted to estimate brain exposure by measuring amounts of the two compounds in primate CSF. Unfortunately, their levels were either undetectable or below the detection sensitivity using our bioanalytical methods (approximately 15 nM for each compound). Therefore, in order to further develop these molecules for clinical testing, additional measurements are required to understand target occupancy, or brain exposures needed for the beneficial (pro-cognitive) effects of these molecules versus their potential for emetic liabilities. Given both the difficulty in estimating the therapeutic index for drugs targeting this mechanism and the potential variability in responsiveness to this class of drugs, a translational non-invasive endpoint to evaluate optimal dosing and obtain evidence of a central effect is desirable. The utilization of PET imaging as a non-invasive approach to estimate occupancy at the target with existing PDE4 non-selective orthosteric ligands would be a challenging task, and currently no PDE4D selective imaging tools exist. Also, given the expected low occupancy required to elicit cognitive effects for this class of compounds in rodents and primates, a PET imaging approach might not be feasible. Other strategies might include the use of pharmaco (ph)-MRI or quantitative (q) EEG techniques, where a relationship between dose and circuitry engagement can be garnered. Overall, based on our results and those of Burgin et al [36], we consider D159687 to be an excellent candidate molecule to conduct this additional work in to assess the potential pro-cognitive effects of PDE4D modulation in neurodegenerative and psychiatric indications.

## Supporting Information

Checklist S1 **ARRIVE Checklist.**
(DOC)

Table S1 **CSF concentration of D159687 following single intravenous administration at 1.0 mg/kg, and on day 1 and day 7 after repeated daily oral administration at 5.0 mg/kg.**
(DOCX)

Table S2 **CSF concentration of D159797 following single intravenous administration at 1.0 mg/kg, and on day 1 and day 7 after repeated daily oral administration at 5.0 mg/kg.**
(DOCX)

Table S3 **Amended dosing schedule (all doses are mg/kg) for evaluation of D159797 after retching and hyper-salivation was noted in animals receiving the highest dose of 5 mg/kg during week 12 (Animals 3017 and 262E) and following 1.5 mg/kg D159797 (animal 6B17) during Week 13.** Additionally, animal 7A5D was excluded from the 1 mg/kg evaluation after observations that the animal did not perform the OR task when dosed at 0.5 mg/kg D159797 (Week 12). However, subsequent evaluations with this animal (unrelated studies; Maccine communication) suggested this was a common occurrence in this animal (occasional disinterest in completing task regardless of treatment) and was deemed to be unlikely related to an adverse event caused by D159797 dosing.
(DOCX)
