# Supplementary material for: Efficacy of Selective PDE4D Negative Allosteric Modulators in the Object Retrieval Task in Female Cynomolgus Monkeys (Macaca fascicularis)
Source: PLoS One. 2014 Jul 22;9(7):e102449. doi: 10.1371/journal.pone.0102449 (PMC4106781; doi:10.1371/journal.pone.0102449)
Supplement: Table S1 — CSF concentration of D159687 following single intravenous administration at 1.0 mg/kg, and on day 1 and day 7 after repeated daily oral administration at 5.0 mg/kg. (DOCX) [file pone.0102449.s002.docx]

|  | **Animal Numbers** | **CSF Collection Time Points (h post dose)** | | | | | | | | | |
| --- | --- | --- | --- | --- | --- | --- | --- | --- | --- | --- | --- |
|  |  | **0** | **0.25** | **1** | **2** | **4** | **6** | **8** | **10** | **12** | **24** |
|  |  |  |  |  |  |  |  |  |  |  |  |
| **IV: 1 mg/kg** | 4377 | QNS | BLQ | BLQ | QNS | QNS | BLQ | BLQ | BLQ | BLQ | BLQ^1^ |
|  | 5264 | QNS | BLQ | QNS | 17.7 | 29.5 | BLQ | BLQ | BLQ^1^ | BLQ | BLQ |
|  | 5271 | QNS | BLQ | BLQ | BLQ | BLQ | BLQ | BLQ | BLQ | BLQ | BLQ |
|  |  |  |  |  |  |  |  |  |  |  |  |
| **PO 5 mg/kg Day 1** | 4377 | BLQ | BLQ | BLQ | BLQ | BLQ | BLQ | BLQ | BLQ | BLQ | BLQ |
|  | 5268 | BLQ | BLQ | BLQ | BLQ | BLQ | BLQ | BLQ | BLQ | BLQ | BLQ |
|  | 5271 | BLQ | BLQ | BLQ | BLQ | BLQ | BLQ | BLQ | BLQ | BLQ | BLQ |
|  |  |  |  |  |  |  |  |  |  |  |  |
| **PO 5 mg/kg Day 7** | 4377 | BLQ | BLQ | BLQ | QNS | BLQ | BLQ | BLQ | BLQ | BLQ | BLQ |
|  | 5268 | BLQ | BLQ | BLQ | BLQ | BLQ | BLQ | BLQ | BLQ | BLQ | BLQ |
|  | 5271 | BLQ | BLQ | BLQ | BLQ | BLQ | BLQ | BLQ | BLQ | BLQ | BLQ |

QNS: Quantity not sufficient for analysis. BLQ: Below level of quantification (13. 7 nM). BLQ^1^ : Below level of quantification (2.73 nM).
