# Supplementary material for: Efficacy of Selective PDE4D Negative Allosteric Modulators in the Object Retrieval Task in Female Cynomolgus Monkeys (Macaca fascicularis)
Source: PLoS One. 2014 Jul 22;9(7):e102449. doi: 10.1371/journal.pone.0102449 (PMC4106781; doi:10.1371/journal.pone.0102449)
Supplement: Table S2 — CSF concentration of D159797 following single intravenous administration at 1.0 mg/kg, and on day 1 and day 7 after repeated daily oral administration at 5.0 mg/kg. (DOCX) [file pone.0102449.s003.docx]

|  | **Animal Numbers** | **CSF Collection Time Points (h post dose)** | | | | | | | | | |
| --- | --- | --- | --- | --- | --- | --- | --- | --- | --- | --- | --- |
|  |  | **0** | **0.25** | **1** | **2** | **4** | **6** | **8** | **10** | **12** | **24** |
|  |  |  |  |  |  |  |  |  |  |  |  |
| **IV: 1 mg/kg** | 5267 | QNS | QNS | BLQ^1^ | BLQ^1^ | BLQ^1^ | BLQ | BLQ | BLQ | BLQ | BLQ |
|  | 5269 | BLQ | BLQ | BLQ | BLQ | BLQ | BLQ | BLQ | BLQ | BLQ | BLQ |
|  | 5277 | BLQ | BLQ | 5.83 | 9.04 | 14.2 | 7.3 | 6.55 | 9.20 | 10.5 | BLQ |
|  |  |  |  |  |  |  |  |  |  |  |  |
| **PO 5 mg/kg Day 1** | 5267 | BLQ | BLQ | BLQ | BLQ | BLQ | BLQ | BLQ | BLQ | BLQ | BLQ |
|  | 5269 | BLQ | BLQ | BLQ | BLQ | BLQ | BLQ | BLQ | BLQ | BLQ | BLQ |
|  | 5277 | BLQ | BLQ | BLQ | BLQ | BLQ | BLQ | BLQ | BLQ | BLQ | BLQ |
|  |  |  |  |  |  |  |  |  |  |  |  |
| **PO 5 mg/kg Day 7** | 5267 | BLQ | BLQ | BLQ | BLQ | BLQ | BLQ | 2.56 | BLQ | BLQ | BLQ |
|  | 5269 | BLQ | BLQ | 9.13 | BLQ | BLQ | BLQ | BLQ | BLQ | BLQ | BLQ |
|  | 5277 | BLQ | BLQ | BLQ | BLQ | BLQ | 4.34 | 4.21 | BLQ | BLQ | BLQ |

QNS: Quantity not sufficient for analysis. BLQ: Below level of quantification (2.48 nM). BLQ^1^ : Below level of quantification (12.4 nM).
