# Supplementary material for: Efficacy of Selective PDE4D Negative Allosteric Modulators in the Object Retrieval Task in Female Cynomolgus Monkeys (Macaca fascicularis)
Source: PLoS One. 2014 Jul 22;9(7):e102449. doi: 10.1371/journal.pone.0102449 (PMC4106781; doi:10.1371/journal.pone.0102449)
Supplement: Table S3 — Amended dosing schedule (all doses are mg/kg) for evaluation of D159797 after retching and hyper-salivation was noted in animals receiving the highest dose of 5 mg/kg during week 12 (Animals 3017 and 262E) and following 1.5 mg/kg D159797 (animal 6B17) during Week 13. Additionally, animal 7A5D was excluded from the 1 mg/kg evaluation after observations that the animal did not perform the OR task when dosed at 0.5 mg/kg D159797 (Week 12). However, subsequent evaluations with this animal (unrelated studies; Maccine communication) suggested this was a common occurrence in this animal (occasional disinterest in completing task regardless of treatment) and was deemed to be unlikely related to an adverse event caused by D159797 dosing. (DOCX) [file pone.0102449.s004.docx]

| **Week** | **Animal** | | | | | | | | **Comments** |
| --- | --- | --- | --- | --- | --- | --- | --- | --- | --- |
|  | **6B17** | **3017** | **3939** | **1A5D** | **7A5D** | **262E** | **5B09** | **OD60** |  |
| **11** | Vehicle PO Monday - Friday | | | | | | | |  |
| **12** | 0.5 | 5 | V | 0.05 | 0.5 | 5 | V | 0.05 | 262E did not attempt OR task - Retching and salivation observed. 3017retched and vomited on immediate completion of OR task. 7A5D did not attempt OR task. |
| **13** | 1.5 | V | 0.05 | 0.5 | 0.05 | V | 0.05 | 0.5 | 6B17 - Mild to moderate retching observed after completion of OR task |
| **14** | V | 0.05 | 0.5 | 1 | V | 0.05 | 0.5 | 1 |  |
| **15** | 0.05 | 0.5 | 1 | V | V | 0.5 | 1 | 0.05 |  |
| **16** | 1 | 1 | V | 0.05 | V | 0.5 | V | 0.05 | (7A5D was not given 1 mg/kg due to reaction of animal to 0.5 mg/kg on Week 12). |
